# Multiple Pathway-Based Genetic Variations Associated with Tobacco Related Multiple Primary Neoplasms

**DOI:** 10.1371/journal.pone.0030013

**Published:** 2012-01-11

**Authors:** Ashwin Kotnis, Junghyun Namkung, Sadhana Kannan, Nallala Jayakrupakar, Taesung Park, Rajiv Sarin, Rita Mulherkar

**Affiliations:** 1 Advanced Centre for Treatment, Research and Education in Cancer, Navi Mumbai, India; 2 Department of Epidemiology and Biostatistics, Case Western Reserve University, Cleveland, Ohio, United States of America; 3 Department of Statistics, College of Natural Science, Seoul National University, Seoul, Korea; Fred Hutchinson Cancer Research Center, United States of America

## Abstract

**Background:**

In order to elucidate a combination of genetic alterations that drive tobacco carcinogenesis we have explored a unique model system and analytical method for an unbiased qualitative and quantitative assessment of gene-gene and gene-environment interactions. The objective of this case control study was to assess genetic predisposition in a biologically enriched clinical model system of tobacco related cancers (TRC), occurring as Multiple Primary Neoplasms (MPN).

**Methods:**

Genotyping of 21 candidate Single Nucleotide Polymorphisms (SNP) from major metabolic pathways was performed in a cohort of 151 MPN cases and 210 cancer-free controls. Statistical analysis using logistic regression and Multifactor Dimensionality Reduction (MDR) analysis was performed for studying higher order interactions among various SNPs and tobacco habit.

**Results:**

Increased risk association was observed for patients with at least one TRC in the upper aero digestive tract (UADT) for variations in *SULT1A1* Arg^213^His, *mEH* Tyr^113^His, *hOGG1* Ser^326^Cys, *XRCC1* Arg^280^His and *BRCA2* Asn^372^His. Gene - environment interactions were assessed using MDR analysis. The overall best model by MDR was *tobacco habit*/*p53*(Arg/Arg)/*XRCC1*(Arg^399^His)/*mEH*(Tyr^113^His) that had highest Cross Validation Consistency (8.3) and test accuracy (0.69). This model also showed significant association using logistic regression analysis.

**Conclusion:**

This is the first Indian study on a multipathway based approach to study genetic susceptibility to cancer in tobacco associated MPN. This approach could assist in planning additional studies for comprehensive understanding of tobacco carcinogenesis.

## Introduction

Tobacco related cancers (TRC) which include carcinoma of lung, esophagus, head-neck, cervix, bladder, stomach, kidney, pancreas, liver and myeloid leukemia account for almost half the global burden of cancer [1], [2]. Tobacco contains a variety of chemical carcinogens which are activated for detoxification by xenobiotic metabolism enzymes (XME). Activated carcinogens can cause DNA damage by forming harmful DNA adducts. The damaged DNA is repaired by elaborate DNA repair machinery. Cells with extensive DNA damage usually undergo apoptosis. Compromise in any of these cellular pathways promotes survival and growth of mutated cells leading to oncogenesis [3].

Genetic susceptibility could be an important determinant in TRC etiology as suggested by familial occurrence of TRC [4]. Identification and characterization of susceptibility factors in common multifactor disorders such as TRC is challenging. This is due to stringent requirement of appropriate samples to analyze the complex gene-environment interactions involved, limitations of conventional statistical methods to reliably determine gene-gene and gene-environment interaction and tools to correlate the genotype to phenotype. Genes important in carcinogenesis are highly polymorphic and contribute to cancer susceptibility. There are numerous large studies associating single nucleotide polymorphisms (SNPs) in a single gene or multiple genes in a single pathway. Often such variants have limited use in assessment of disease risk, since most of the variants have low penetrance and confer a relatively small risk. Multipathway based association study should help to identify a cumulative effect of low penetrance alleles in predisposition [5]. Consensus has failed to emerge regarding the combination of genetic alterations that drives tobacco carcinogenesis. There is no report yet on a multipathway based approach to study genetic susceptibility to cancer in tobacco associated multiple primary neoplasms (MPN).

**Figure 1:**
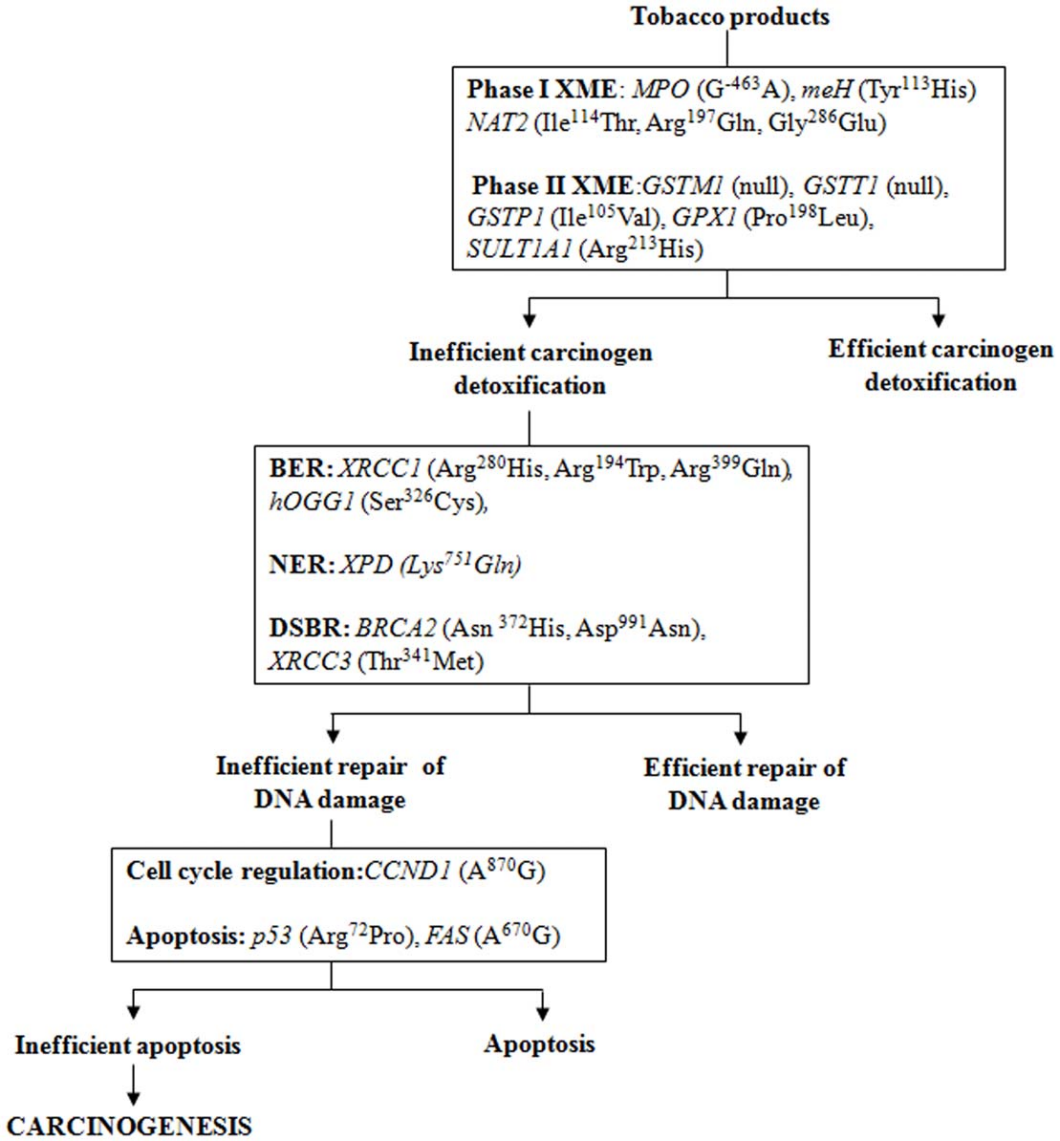
Low penetrance effect of SNPs in carcinogenesis.

In the present case-control study, we have investigated the hypothesis that cumulative effect of low penetrance alleles predispose to tobacco induced MPN (Fig. 1). It is believed that patients with MPN provide a genetically enriched resource to study predisposition to cancer [6]. Association of 21 candidate SNPs in 18 genes from pathways of xenobiotic metabolism, DNA repair, cell cycle regulation and apoptosis implicated in tobacco carcinogenesis was studied in a unique group of individuals with tobacco related MPN. The risk association was analyzed using logistic regression and higher order genetic interactions were studied using multifactor dimensionality reduction (MDR) analysis.

## Results

In the present study 151 cases with MPN and 210 cancer-free controls were analyzed. Using IARC definition of Tobacco Related Cancer (TRC) these 151 MPN patients were further sub classified as patients with at least one TRC in the UADT (n = 113), none of the TRC in the UADT (n = 17) and those with no TRC (n = 21; Table 1). In all the categories, majority of the cancers were metachronous, that is, the second primary cancer was diagnosed 6 months or more after diagnosis of first cancer.

**Table 1:** Demographics of study subjects.

Category	TRC inside UADT (n = 113) (%)	TRC outside UADT (n = 17) (%)	None TRC (n = 21) (%)	Cancer free controls (n = 210) (%)
Males	75 (66)	3 (18)	6 (29)	137 (65)
Females	38 (34)	14 (82)	15 (71)	73 (34)
Age(years)				
Median	50	50	44	46
Range	26–75	31–70	23–79	20–84
Types of MPN				
Synchronous	33 (29)	3 (18)	3 (14)	NA
Metachronous	80 (71)	14 (82)	18 (86)	NA
Tobacco Habit				
No habit	15 (13)	8 (47)	11 (52)	21 (10)
Only T	70 (62)	8 (47)	8 (38)	160 (76)
T+A	25 (22)	1 (6)	1 (5)	27 (13)
No information	3 (3)	0	1 (5)	2 (1)

T- Tobacco habit alone either in form of chewing or smoking.

T+A – Tobacco in form of chewing or smoking along with alcohol.

48.6% cases and 61.4% controls used smokeless tobacco in form of masheri.

Majority of the patients had both cancers in the UADT region. Patients and controls were mainly from North India, with tobacco habit. Among the tobacco users, 48.6% cases and 61.4% controls used smokeless tobacco which was in the form of application of roasted tobacco (masheri) over gums or chewing a mix of tobacco with one or more ingredients like lime, betel nut or betel leaf. The quanta of tobacco consumed were not available for many subjects as it was self reported information.

The genotype distribution of SNPs was compared in cases using dominant model (homozygous wild type versus homozygous variant+heterozygous) and extreme model (homozygous wild type versus homozygous variant). Selection of these models was done on the biological plausibility that homozygous and heterozygous variant conferred risk compared to wild type.

Amongst the 21 SNPs selected from genes in different pathways, univariate analysis showed risk association in a few genes as shown in Table 2 and Table S1. Crude OR and OR adjusted to age and gender were considered for statistically significant association. Increased risk association was observed for patients with at least one TRC in the UADT category for xenobiotic metabolizing genes, *SULT1A1* Arg^213^His (Extreme model, OR = 6.6, 95% CI 1.47–29.34; Dominant model, OR = 1.74, 95% CI 1.07–2.82), *meH* Tyr^113^His (Extreme model, OR = 2.55, 95% CI 1.19–5.47), DNA repair genes, *hOGG1* Ser^326^Cys (Dominant model, OR = 1.81, 95% CI 1.12–2.90; Heterozygous OR = 1.91, 95% CI 1.16–3.15), *XRCC1* Arg^280^His (Dominant model, OR = 1.80, 95% CI 1.06–3.08), BRCA2 (Heterozygous OR = 1.93, 95% CI 1.11–3.35). In the TRC outside group significant risk association was observed for xenobiotic metabolizing genes *MPO* G −463 A (Extreme model, OR = 8.06, 95% CI 2.16–30.72; Dominant model, OR = 3.66, 95% CI 1.37–9.79), *SULT1A1* Arg^213^His (Extreme model, OR = 6.92, 95% CI 2.27–20.96; Heterozygous, OR = 7.15, 95% CI 2.34–21.67), protective association was observed for the cell cycle regulating gene *Cyclin D1* A^870^G (Extreme model OR = 0.27, 95%CI 0.09–0.723; Heterozygous, OR = 0.26, 95%CI 0.09–0.82).

**Table 2:** Univariate analysis of SNP with significant effects.

Pathway	Gene	Polymorphism (SNP ID)	Biological Effect	Type of variation		Odds Ratio (95% CI)			
					Controls/Atleast one in UADT/TRC outside UADT	At least one in UADT	P value	TRC outside UADT	P value
Xenobiotic metabolism	*MPO*	G^−463^ APromoter region(rs 2333227)	Decreased expression and detoxification	**GG**	141/70/7	-		-	
				GA	45/34/6	1.52(0.87–2.67)	0.131	2.686(0.752–9.506)	0.100
				AA	10/9/4	1.81(0.64–5.104)	0.218	*8.06(1.639–38.837)	0.008
				GA+AA	55/43/10	1.58(0.94–2.65)	0.076	*3.66(1.207–11.328)	0.013
	*SULT1A1*	Arg^213^His(rs9282861)	G>A, Decreased enzyme activity, thermostability	**Arg/Arg**	132/60/4	-		-	
				Arg/His	60/43/13	1.58(0.93–2.67)	0.075	*7.15(2.053–27.244)	<0.001
				His/His	2/6/0	*6.6(1.16–48.85)	0.017	0(0–199.144)	1.000
				Arg/His+His/His	62/49/13	*1.74(1.07–2.82)	0.026	*6.92(1.99–26.336)	<0.001
	*mEH*	Tyr^113^His(rs1051740)	Decreased detoxification	**Tyr/Tyr**	78/25/6	-		-	
				Tyr/His	95/53/6	1.74(0.96–3.18)	0.054	0.82(0.223–3.019)	0.772
				His/His	22/18/3	*2.55(1.11–5.92)	0.024	1.77(0.319–8.970)	0.426
				Tyr/His+His/His	117/71/9	*1.89(1.07–3.365)	0.020	1.00(0.310–3.314)	1.000
DNA Repair	*hOGG1*	Ser^326^Cys(rs1052133	Altered localization	**Ser/Ser**	114/49/7	-		-	
				Ser/Cys	62/51/7	*1.91(1.13–3.25)	0.011	1.84(0.548–6.170)	0.387
				Cys/Cys	14/8/3	1.33(0.47–3.66)	0.624	3.49(0.628–17.735)	0.108
				Ser/Cys+Cys/Cys	76/59/10	*1.81(1.09–3.00)	0.016	2.14(0.712–6.572)	0.198
	*XRCC1*	Arg^280^His(rs25489)	Defective localization, decreased repair	**Arg/Arg**	157/80/14	-		-	
				Arg/His	33/30/2	1.78(0.98–3.25)	0.055	0.680(0.101–3.371)	1.000
				His/His	4/4/1	1.96(0.40–9.63)	0.451	2.804(0.11–30.328)	0.363
				Arg/His+His/His	37/34/3	*1.80(1.02–3.20).	0.036	0.91(0.196–3.632)	1.000
	*BRCA2*	Asn^372^His(rs144848)	Reduced DNA repair	**Asn/Asn**	81/30/5	-		-	
				Asn/His	70/50/6	*1.93(1.07–3.49)	0.027	1.389(0.355–5.529)	0.757
				His/His	35/14/4	1.08(0.48–2.43)	0.850	1.851(0.388–8.608)	0.459
				Asn/His+His/His	105/64/10	1.65(0.95–2.870)	0.070	1.543(0.461–5.424)	0.590
Cell Cycle regulation	*Cyclin D1*	A ^870^ GSplice site(rs 603965)	Nuclear accumulation of protein	**GG**	67/33/11				
				GA	93/55/4	1.20(0.68–2.12)	0.589	*0.262(0.067–0.943)	0.028
				AA	45/27/2	1.22(0.62–2.41)	0.627	0.271(0.039–1.392)	0.129
				GA+AA	138/82/6	1.21(0.71–2.05)	0.530	*0.265(0.083–0.817)	0.015

We have conducted MDR analysis accounting for missing values [7] and the one-way to five-way interaction models were considered. As shown in Table 3, the overall best model across one to five-way interaction models was *habit*/*p53*(Arg^72^Arg)/*XRCC1*(Arg^399^His)/*meH*(Tyr^113^His) that had maximum CVC (Cross validation consistency) and maximum test accuracy (CVC = 8.3; test accuracy = 0.69). To obtain effect size of individual genotype combination, OR MDR analysis [8] was conducted for this four-way interaction model as shown in Table S2. Because the number of individuals for each combination of genotype and habit was relatively small, only one variable combination Habit = 1, *p53* (Arg^72^Arg) = 0, *XRCC1* (Arg^399^His) = 1, and *meH* (Tyr^113^His) = 1 had odds ratio with significant confidence intervals (OR = 3.217; 95% CI 1.201–10.177).

**Table 3:** MDR analysis.

*Model*	*CVC*	*train accuracy*	*test accuracy*
Habit	7.6	0.637	0.612
Habit/*Cyclin D1* (A^870^G)*	4.2	0.667	0.645
Habit/*SULT1A1* (Arg^213^His)/*XRCC1* (Arg^399^His)	5.6	0.723	0.654
**Habit/** ***p53*** ** (Arg^72^Arg)/** ***XRCC1*** ** (Arg^399^His)/** ***mEH*** ** (Tyr^113^His)**	**8.3**	**0.778**	**0.69**
*SULTA1* (Arg^213^His)/*p53* (Arg^72^Pro) *XRCC1* (Arg^399^His)/*BRCA2* (Asn^372^His)/*mEH* (Tyr^113^His)	7.8	0.855	0.632

Analysis has been repeated 10 times after shuffling the order of individuals and the mean of evaluation measures are presented. The results of the best model are in bold.

*Habit/*SULT1*A1 (Arg^213^His), *SULT1*A1 (Arg^213^His)/*BRCA2* (Asn^372^His) and Habit/*Cyclin D1* (A^870^G) have been selected 1, 4, and 5 times out of 10 repeated analyses, respectively.

All the 21 SNPs were analyzed for HWE of which 12 SNPs were in HWE for the control group where as 9 SNP were not in HWE. However, all the 3 SNPs (*p53* (Arg^72^Arg)/*XRCC1* (Arg^399^His)/*mEH* (Tyr^113^His) which showed significant association together with tobacco habit in the MDR analysis were in HWE. SNPs with significant association also showed HWE in controls group. We perfomed linkage disequilibrium analysis for variants in *XRCC1*, *NAT2* and *BRCA2* and observed significant association between *NAT2 Ile^114^Thr* and *NAT2 Arg^197^Gln*, *NAT2 Ile^114^Thr* and *NAT2 Gly^286^Glu*, *BRCA2 Asp^991^Asn* and *BRCA2 Asn^372^His* as shown in Table S3.

## Discussion

In the present case-control study we have examined a set of biologically plausible SNPs implicated in tobacco carcinogenesis (Fig. 1). Risk association of these SNPs for tobacco related cancers has been investigated by using conventional statistics and through MDR analysis. Though not a consistent finding, each of the SNP identified by us, and a large number of other SNPs have been shown to be associated with tobacco related cancers in previous case-control studies or their meta-analyses [1], [9], [10], [11], [12], [13]. The evidence for cumulative effect of various genetic alterations on metabolic and cellular pathways involved in tobacco carcinogenesis although compelling [5], is based on piecemeal evidence from heterogeneous studies of single or few related SNPs, in a background of large number of genetic and environmental risk modifiers. Only few genome-wide association studies (GWAS) have been conducted for tobacco related cancers so far and these are yet to provide major leads in tobacco carcinogenesis [14].

It is emerging that for an unbiased qualitative and quantitative assessment of gene-gene and gene-environment interactions, clinically relevant insight in tobacco carcinogenesis may not come from additional studies confirming or refuting risk association of known SNPs, but through exploration of alternative research strategies, model systems and analytical methods. Towards this goal we have incorporated three distinct elements in our study. Firstly, we have adopted a biologically holistic approach of examining SNPs in the key genes of major pathways in tobacco carcinogenesis. We have chosen a biologically enriched clinical model system of tobacco related multiple primary neoplasms (MPN-TRC). We had earlier hypothesized [5], [10], [15], [16] that individuals who develop tobacco related MPN, represent a cohort of individuals with enhanced gene-environment and gene-gene interaction. Only recently, the unique biological and statistical utility of MPN in molecular epidemiological studies has been highlighted by others [6]. These authors provide empirical evidence that for MPN of the same organ, the relative risk is approximately the square of the relative risk as found in the traditional case-control studies using single primary cancers. In our study, 75% of the TRC MPN were within the UADT, which is one continuous epithelial lining exposed to tobacco carcinogens.

The third aspect of our study is the statistical approaches used. Studying higher order gene interactions using logistic regression is laborious and has low statistical power due to very high degrees of freedom. Hence we used multifactor dimensionality reduction (MDR) as a complementary statistical approach for studying higher order interactions among various SNPs analyzed and tobacco habit. This combination of testing multiple SNPs using MDR showed 4 factor model of *tobacco habit*, *p53* (Arg^72^Arg), *XRCC1* (Arg^399^His) and *mEH* (Tyr^113^His) with an OR of 3.217 (95% CI: 1.2–10.18) and cross validation consistency of 8.3 as strongest risk predictor to MPN. Cross validation consistency refers to the number of times a particular interaction model is selected across 10 cross-validation datasets and the best test accuracy of 0.69. We have taken several approaches to control for false positive findings which may emerge due to multiple testing. We further tested the 2–4 MDR genetic models using logistic regression and found significant association of the models with tobacco related MPN. The SNPs with significant association in univariate analysis also showed significant interaction in MDR. The advantage of this observation is that MDR makes no assumption about the data distribution and does not require correction of multiple testing, which is helpful for studies with small sample size. The controls were matched for ethnicity and tobacco habit reducing the confounding risk due to ethnicity.

**Figure 2:**
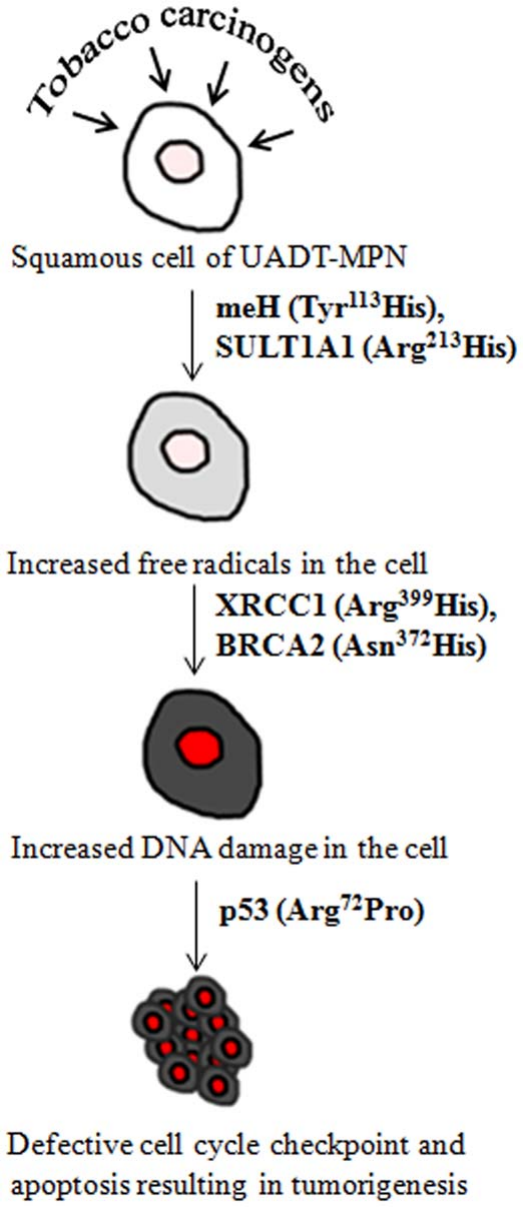
Cumulative effect model of polymorphisms predisposing to tobacco related cancers.

It is biologically plausible that decreased detoxification due to variant *meH* Tyr^113^His results in increased DNA damage which is inefficiently repaired by the base excision repair protein XRCC1 (Fig. 2). Presence of Arg^399^Gln SNP in an evolutionarily conserved region of *XRCC1*, expression of meH, XRCC1 in mucosa of the upper aerodigestive tract [17], [18], protein interaction of BRCA2 and p53 [19] strengthen this model.

Meta-analysis has shown varying effects of *XRCC1* Arg^280^His in tobacco related cancers [1], [9]. Studies in Chinese lung cancer (n = 108) and Korean gastric cancer population (n = 172) showed significant risk association with Arg/His or His/His genotype [1], [20]. An Indian study [21] on oral cancer observed marginal risk conferred by His/His. Some studies reported no association of Arg^280^His with esophageal [22], bladder [23], gastric [24], [25] and lung cancer [1] although a few studies showed protection by His/His genotype in lung cancer [26], [27]. We observed stronger risk association of Arg/His in the patient population with at least one cancer in UADT-MPN group. Ethnic differences could be one of the determining factors in risk association, as mean frequency of His/His reported in Asian population is 13% (range 3–36%) where as in Caucasians it is 36% (range 2–47%) [10].

The variant *meH* Tyr^113^His which results in 30–50% decreased enzyme activity [28] has been significantly associated with cancers of larynx and lung [11], [12], [29]. Other studies observed no association with lung [30], head and neck [31] and laryngeal cancers [26]. Most of the studies show trend towards risk for this polymorphism and it is probable that the risk effect was enriched in combination with other genes in the MPN population.

Dominant and heterozygous models showed significant risk association of *hOGG1* Ser^326^Cys SNP in UADT-TRC group. Studies have reported contrasting observation on risk association of this SNP in UADT cancers. Recently published Indian studies [27], [32] showed significant protective effect in head-neck cancer and no association of the *hOGG1* Cys/Cys and Ser/Cys variant, whereas meta-analysis and another study [1] observed risk associaton of Cys/Cys genotype in lung cancer. Biochemical evidences support Cys/Cys genotype as risk conferring genotype due to lower protein activity compared to Ser/Ser variant, observed in head-neck [33] and lung cancer [34].

The *BRCA2* Asn^372^His SNP showed significant risk association for dominant model for at least one TRC in UADT category and trend for risk in other models in at least one TRC in UADT and TRC outside UADT categories. The variation is located in the conserved region of the *BRCA2* gene. Not much is known about the functional role of this SNP and its association in tobacco related cancers. The SNP has been associated with breast cancers [35] lymphoma [36], not associated with lung cancer [13], [37]. *BRCA2* Asn^372^His showed higher sensitivity to gamma radiation along with other polymorphisms in the DNA repair pathway [13].

There are conflicting reports on the risk association of the *p53* Arg^72^Pro SNP. While most studies report a weak protective association or no association of the p53 wild type Arg^72^Arg genotype for various cancers [38], [39] in our MDR model the Arg/Arg genotype in combination with the other two genotypes and tobacco, was associated with the risk of tobacco related MPNs. Several other studies have shown similar risk association between the wild type Arg/Arg genotype and breast [40], gastric [41], head and neck [42] and colorectal cancers [43], [44]. It may however be noted that studies which examined the gene-gene or gene environment interactions, the protective effect of the *p53* Arg^72^Pro variant allele was seen in combination with other genotypes like the p53 intron 6 diplotype for head and neck cancers [42] and gastric cancer [41] or with the use of non steroidal anti-inflammatory drugs in colorectal cancer [44].

Despite the strengths and biological plausibility of the associations observed in our study, there are inherent limitations. Reliable estimation on the quanta of tobacco and alcohol consumed was not available as it was based on self-reported information. It is quite likely that several other important gene – gene and gene environment interactions exist that have not been evaluated in our study. It is also possible some of the SNPs studied and their interactions failed to emerge as significant risk association due to the limited sample size.

This is the first study to examine key SNPs in major metabolic and biological pathways implicated in tobacco carcinogenesis in the unique Indian MPN population. This study supports MPN to be an enriched model to predict cumulative genetic interactions. We anticipate the relevance of correlating the cumulative effect of variant genotypes to cellular phenotype in response to tobacco carcinogens. More importantly, for tobacco carcinogenesis it is difficult to quantify the redundancy of individual SNP, genes and pathways and this may vary in different geo-ethnic groups due to significant differences in the frequency of specific SNPs and or exposure to environmental, dietary co-carcinogens and protective agents. However, our approach to examine the multi-pathway tobacco carcinogenesis incorporates large body of research findings in a genetically enriched clinical model. Our approach could complement the GWAS approach by testing the leads provided by high quality GWAS studies.

## Materials and Methods

### Ethics Statement

Approval from Hospital Ethics Committee, Tata Memorial Centre, Mumbai was obtained before starting the study. Blood was collected after obtaining written informed consent from patients as well as healthy donors.

### Study Population

Genotyping was carried out on 151 consecutive multiple primary neoplasm (MPN) patients. The cases were accrued from a registry of patients with MPN or familial cancers established at the Tata Memorial Hospital, Mumbai, in 1996 by one of the authors (RS). All the cases had histological or cytological confirmation of the primary cancer and each of the cancers was classified as TRC or non-TRC as per the IARC criteria [2]. There was no restriction for age at diagnosis, gender or carcinogen exposure. For defining two cancers as distinct multiple primaries, modified Hong's criteria [45] was used, which states that (a) there is >2 cm of normal intervening mucosa between two primaries in head and neck region; (b) lung as second primary if present, should be of different histology, or be solitary and with characteristic radiology of lung cancer; and (c) there is no evidence of haematogenous spread. Bilateral cancers in paired organs such as breast, ovaries or kidneys were not classified as MPN. Majority of the MPN cases in the registry were from the western and northern parts of India.

The cancer-free controls (n = 210) were volunteers who consented to donate blood or buccal washes for the study and were of similar geo-ethnic background as the cases. They were either visiting our hospital in the Preventive Oncology Department for cancer screening (n = 131) or visiting government dental college for various non-malignant, dental ailments (n = 73). A few controls were healthy, ethnically matched workers from Mumbai (n = 6). A majority of them were tobacco users (89%).

Detailed questionnaire including ethnicity and lifetime history of tobacco and alcohol use was obtained from all cases and controls. Family history of cancer was obtained from majority of MPN cases and cancer-free controls. After obtaining informed consent, 3–6 ml of peripheral blood was collected from each subject. Exfoliated buccal cells (mouthwash samples) were collected in sterile phosphate buffered saline from control individuals who were reluctant to give blood (n = 79). The study was approved by the Hospital Ethics Committee, Tata Memorial Centre, Mumbai.

### DNA extraction and genotyping

Genomic DNA was extracted from peripheral blood/mouthwash samples using phenol chloroform method standardized in our laboratory [46]. Genotyping was done either by PCR-RFLP (Restriction fragment length polymorphism) or by SNaPshot method (ABI, USA). Primer sequences for PCRs were obtained from published literature and the conditions for PCR were standardized. The primer sequence, PCR conditions and restriction enzymes used for RFLP are available upon request. PCR was done in 96-well thermal cycler (ABI) in 25 µL volume containing PCR buffer (Invitrogen), 0.2 mmol/L deoxynucleotide triphosphates (Invitrogen), 0.5 mmol/L MgCl_2_ (Invitrogen), 0.25 unit Taq-Polymerase (Invitrogen), and 40 ng template DNA.

The authenticity of the PCR products was confirmed by sequencing at least five PCR products at random on an automated DNA sequencer (ABI Prism 3100 Avant) using the Big Dye terminator kit (ABI Prism, Foster City, CA, USA) as per the manufacturer's instructions.

### Multiplex genotyping

Seven polymorphisms (*NAT1*, *NAT2*, *BRCA1*, *BRCA2*, *GPX*, *meH* and *NAT3*) were genotyped by multiplex PCR using SNaPshot. The assay was performed using SNaPshot ready reagent kit (ABI, USA). To the ready reagent, SBE primers (0.3 ρmol), EXO-SAP purified PCR products were added in a total reaction volume of 5 µl and incubated for 25 cycles of 96°C for 10 seconds, 50°C for 5 seconds and 60°C for 30 seconds. After the reaction, samples were purified by incubating with SAP (0.5 U) 37°C for 60 minutes followed by 75°C for 15 minutes. The purified products were run by capillary electrophoresis performed in 96 well plates in the ABI Prism™ 3100 genetic analyzer and analyzed using the Genemapper software (version 3.5). For SNP detection, the post purification products were denatured with de-ionized formamide and Genescan™ 120 Liz® size standard (Applied Biosystems) as per the manufacturers instructions at 95°C for 5 minutes followed by instant chilling on ice prior to loading on to the Sequencer. The electropherograms were depicted as two coloured peaks corresponding to two alleles for each heterozygous marker (SNP) or of one coloured peak for homozygous samples. As the fragments for each SNP are of varying sizes, the peaks did not overlap. To assure distinct recognition of closely lying peaks and avoid any chance of overlapping, the SNPs were grouped into two distinct panels based on the fragment size using the Primer Focus software (ABI). The software analyzed the genotypes according to the size and position of the alleles and accepted alleles that fall into the predetermined panel and represented genotypes of the entire sample set in a readily usable excel format.

### Statistical Analysis

#### Univariate analysis

Hardy Weinberg Equilibrium in the healthy controls was evaluated using χ^2^ test. Crude odds ratio and 95 percent confidence intervals were calculated for univariate analysis. For risk estimation the genotypes were a priori classified as homozygous low-risk or high-risk alleles based on their function in respective pathway. For each SNP, the Odds Ratio (OR) with its 95% Confidence Interval (CI) was estimated for the variant allele in its heterozygous and homozygous form taking the wild type homozygous allele as reference. Two sided p values were reported and considered significant if p<0.05.

#### Statistical analysis of gene-gene/gene-environment interactions

In order to analyze interactions between SNPs and between SNPs and tobacco habits contributing to cancer risk, multifactor dimensionality reduction (MDR) approach was used. MDR is a non-parametric and genetic model free gene-gene interaction analysis method. This method had been proposed to overcome limitation of logistic regression in the analysis of high order interaction models where sparse data occur frequently [47], [48]. To account for individual data with missing values, ‘*Available*’ MDR approach was adopted in the analysis and analysis was performed using impute MDR in R packages [7]. *Available* MDR approach uses all the individuals who have complete data for a set of SNPs or habit variable that are included in a considered interaction model, thus it uses different number of individuals for each of possible interaction models. In the analysis of gene-gene or gene-environment interactions, individuals with more than 5 missing values were excluded. The analysis was repeated 10 times after shuffling the order of individuals and average of cross-validation consistency (CVC), training and test accuracies are presented. CVC is defined as the number of times a particular interaction model is selected across 10 cross-validation datasets. For the final selected model, we conducted odds ratio based MDR analysis (OR MDR) [8] to get the individual genotype effects.

## Supporting Information

Table S1 **Univariate analysis of SNPs which do not show significant effects.**
(DOC)

Table S2 **The OR MDR analysis results for the final best model.** The odds ratios having significant asymptotic confidence interval are in bold.
(DOC)

Table S3 **Linkage Disequilibrium (LD) analysis.**
(DOC)
