# Supplementary material for: Multiple Pathway-Based Genetic Variations Associated with Tobacco Related Multiple Primary Neoplasms
Source: PLoS One. 2012 Jan 11;7(1):e30013. doi: 10.1371/journal.pone.0030013 (PMC3256192; doi:10.1371/journal.pone.0030013)
Supplement: Table S1 — Univariate analysis of SNPs which do not show significant effects. (DOC) [file pone.0030013.s001.doc]

**Supplementary Table S1: Univariate analysis of SNPs which do not show significant effects.**

| **Pathway** | **Gene** | **Polymorphism (SNP ID)** | **Biological Effect** | **Type of variation** | **Controls/ Atleast one in UADT/TRC outside UADT** | **At least one in UADT**  **Odds Ratio (95% CI)** | **P value** | **TRC outside UADT**  **Odds Ratio (95% CI)** | **P value** |
| --- | --- | --- | --- | --- | --- | --- | --- | --- | --- |
| DNA Repair | *BRCA2* | Asp ^991^ Asn (rs 179943) | Reduced DNA repair capacity | **Asp/Asp**  Asp/Asn  Asn/Asn  Asp/Asn+Asn/Asn | 112/51/10  69/38/4  20/10/1  89/48/5 | -  1.209(0.698-2.093)  1.098(0.442-2.690)  1.184(0.710-1.975) | 0.509  0.833  0.538 | -  0.649(0.164-2.371)  0.560(0.025-4.701)  0.629(0.180-2.098) | -  0.575  1.000  0.591 |
|  | *XRCC1* | Arg ^194^ Trp  (rs 1799782) | Decreased repair capacity | **Arg/Arg**  Arg/Trp  Trp/Trp  Arg/Trp+Trp/Trp | 159/86/13  32/21/4  3/4/0  35/25/4 | -  1.213(0.631-2.327)  2.465(0.463-14.243)  1.321(0.713-2.442) | 0.532  0.252  0.371 | -  1.529(0.391-5.500)  0(0-31.060)  1.398(0.36-4.996) | -  0.504  1.000  0.526 |
|  |  | Arg 399 Gln  (rs 25487) | Decreased repair capacity | **Arg/Arg**  Arg/Gln  Gln/Gln  Arg/Gln + Gln/Gln | 63/53/7  92/46/9  26/16/1  118/62/10 | -  0.594(0.346-1.020)  0.731(0.333-1.596)  0.625(0.377-1.035) | 0.053  0.469  0.067 | -  0.880(0.281-2.792)  0.346(0.015-3.070)  0.763(0.252-2.351) | -  0.797  0.437  0.604 |
|  | *XPD* | Lys ^751^ Gln (rs 13181) | Decreased repair capacity | **Lys/Lys**  Lys/Gln  Gln/Gln  Lys/Gln+Gln/Gln | 79/46/6  102/53/8  16/15/2  118/68/10 | -  0.892(0.529-1.504)  1.610(0.678-3.822)  0.990(0.602-1.628) | -  0.706  0.304  1.000 | -  1.033(0.309-3.521)  1.646(0.208-10.424)  1.116(0.355-3.618) | -  1.000  0.626  1.000 |
|  | *XRCC3* | Thr ^241^ Met  (rs 861539) | Higher DNA adduct formation | Thr/Thr Thr/Met  Met/Met  Thr/Met+Met/Met | 10/9/0  71/34/6  100/65/11  171/99/17 | -  0.532(0.178-1.590)  0.722(0.254-2.063)  0.643(0.232-1.792) | -  0.294  0.622  0.462 | -  inf(0.125-inf)  inf(0.180-inf)  inf(0.171-inf) | 1.000  0.596  1.000 |
| Apoptosis | *p53* | Arg ^72^ Pro  (rs 1042522) | Decreased apoptosis | **Arg/Arg**  Arg/Pro  Pro/Pro  Arg/Pro+Pro/Pro | 50/33/6  89/61/8  50/19/3  139/80/11 | -  1.038(0.580-1.863)  0.909(0.451-1.830)  0.872(0.503-1.514) | -  1.000  0.869  0.690 | -  0.749(0.219-2.605)  0.500(0.093-2.431)  0.659(0.211-2.129) | 0.772  0.490  0.409 |
|  | *FAS* | A 670 G  (Enhancer region)  (rs 1800682) | Decreased apoptosis | GG AG  AA AG+AA | 41/20/5  46/34/3  11/11/2  57/45/5 | -  1.515(0.715-3.223)  2.050(0.681-6.209)  1.618(0.793-3.315) | -  0.295  0.200  0.187 | -  0.535(0.094-2.798)  1.491(0.172-10.786)  0.719(0.166-3.113) | -  0.477  0.643  0.741 |

| Xenobiotic metabolism | *GSTM1* | Nil  (NA) | Deletion, no protein, decreased detoxification | Not Null Null | 146/79/11  62/38/6 | -  1.133(0.675-1.898) | -  0.619 | -  1.284(0.402-3.975) | -  0.596 |
| --- | --- | --- | --- | --- | --- | --- | --- | --- | --- |
|  | *GSTT1* | Nil  (NA) | Deletion, no protein, decreased detoxification | Not NullNull | 168/87/16  32/28/0 | -  1.690(0.920-3.102) | -  0.075 | -  0(0-1.755) | -  0.137 |
|  | *GSTP1* | Ile ^105^ Val  (rs1695) | Decreased detoxification, affects thermostability | Ile/Ile Ile/Val  Val/Val  Ile/Val+Val/Val | 109/70/11  63/36/6  19/4/0  82/40/6 | -  0.890(0.519-1.525)  0.328(0.09-1.081)  0.760(0.455-1.266) | -  0.700  0.064  0.275 | -  0.944(0.294-2.935)  0(0-2.983)  0.725(0.228-2.234) | -  1.000  0.361  0.616 |
|  | *GPX1* | Pro ^198^ Leu  (rs1050450) | Decreased enzyme activity, decreased detoxification | Pro/Pro Pro/Leu  Leu/Leu  Pro/Leu+Leu/Leu | 119/70/8  37/16/4  32/9/3  69/25/7 | -  0.735(0.361-1.484)  0.478(0.199-1.121)  0.616(0.344-1.098) | -  0.418  0.072  0.084 | -  1.608(0.381-6.364)  1.395(0.275-6.272)  1.509(0.468-4.834) | -  0.489  0.705  0.580 |
|  | NAT2 | Ile ^114^ Thr  (rs1801280) | Decreased enzyme activity, decreased detoxification | Ile/Ile Ile/Thr  Thr/Thr  Ile/Thr+Thr/Thr | 72/34/5  108/51/7  20/12/3  128/63/10 | -  1.000(0.571-1.752)  1.271(0.514-3.119)  1.042(0.609-1.787) | 1.000  0.669  0.898 | -  0.933(0.253-3.547)  2.160(0.369-11.747)  1.125(0.336-3.953) | -  1.000  0.380  1.000 |
|  |  | Arg ^197^ Gln  (rs1799930) |  | Arg/Arg Arg/Gln  Gln/Gln Arg/Gln + Gln/Gln | 67/30/8  115/57/6  19/10/1  134/67/7 | -  1.107(0.627-1.959)  1.175(0.445-3.070)  1.117(0.643-1.945) | -  0.786  0.821  0.695 | -  0.437(0.128-1.464)  0.441(0.019-3.904)  0.438(0.136-1.399) | -  0.158  0.679  0.159 |
|  |  | Gly ^286^ Glu  (rs1799931) |  | Gly/Gly Gly/Glu  Glu/Glu  Gly/Glu+Glu/Glu | 174/86/11  22/10/3  2/0/0  24/10/3 | -  0.920(0.387-2.150)  0(0-8.395)  0.843(0.358-1.949) | -  1.000  1.000  0.846 | -  2.157(0.439-9.321)  0(0-73.441)  1.977(0.405-8.473) | -  0.223  1.000  0.396 |
