# Supplementary material for: Multiple Pathway-Based Genetic Variations Associated with Tobacco Related Multiple Primary Neoplasms
Source: PLoS One. 2012 Jan 11;7(1):e30013. doi: 10.1371/journal.pone.0030013 (PMC3256192; doi:10.1371/journal.pone.0030013)
Supplement: Table S2 — The OR MDR analysis results for the final best model. The odds ratios having significant asymptotic confidence interval are in bold. (DOC) [file pone.0030013.s002.doc]

**Supplementary Table S2:** The OR MDR analysis results for the final best model.

The odds ratios having significant asymptotic confidence interval are in bold.

| X1 | X8 | X10 | X20 | Case freq | Control freq | Hi/Low | Odds ratio | 95% confidence intervals | |
| --- | --- | --- | --- | --- | --- | --- | --- | --- | --- |
| 1 | 0 | 0 | 0 | 1 | 6 | Low | 0.238 | 0.106 | 1.951 |
| 2 | 0 | 0 | 0 | 1 | 0 | High | Inf | - | Inf |
| 0 | 1 | 0 | 0 | 1 | 0 | High | Inf | - | Inf |
| 1 | 1 | 0 | 0 | 9 | 6 | High | 2.145 | 0.957 | 5.848 |
| 2 | 1 | 0 | 0 | 5 | 2 | High | 3.575 | 0.891 | 18.084 |
| 0 | 2 | 0 | 0 | 1 | 0 | High | Inf | - | Inf |
| 1 | 2 | 0 | 0 | 2 | 6 | Low | 0.477 | 0.213 | 2.317 |
| 0 | 0 | 1 | 0 | 1 | 0 | High | Inf | - | Inf |
| 1 | 0 | 1 | 0 | 1 | 5 | Low | 0.286 | 0.118 | 2.413 |
| 2 | 0 | 1 | 0 | 1 | 2 | Low | 0.715 | 0.178 | 7.785 |
| 0 | 1 | 1 | 0 | 1 | 0 | High | Inf | - | Inf |
| 1 | 1 | 1 | 0 | 2 | 9 | Low | 0.318 | 0.164 | 1.442 |
| 2 | 1 | 1 | 0 | 0 | 5 | Low | 0 | - | - |
| 0 | 2 | 1 | 0 | 1 | 0 | High | Inf | - | Inf |
| 1 | 2 | 1 | 0 | 1 | 7 | Low | 0.204 | 0.097 | 1.636 |
| 2 | 2 | 1 | 0 | 0 | 1 | Low | 0 | - | - |
| 1 | 0 | 2 | 0 | 1 | 0 | High | Inf | - | Inf |
| 1 | 1 | 2 | 0 | 0 | 4 | Low | 0 | - | - |
| 2 | 1 | 2 | 0 | 1 | 0 | High | Inf | - | Inf |
| 1 | 2 | 2 | 0 | 0 | 5 | Low | 0 | - | - |
| 1 | 0 | 0 | 1 | 4 | 11 | Low | 0.52 | 0.285 | 1.59 |
| 2 | 0 | 0 | 1 | 2 | 0 | High | Inf | - | Inf |
| 0 | 1 | 0 | 1 | 1 | 1 | High | 1.43 | 0.201 | 22.611 |
| 1 | 1 | 0 | 1 | 6 | 14 | Low | 0.613 | 0.359 | 1.544 |
| 2 | 1 | 0 | 1 | 1 | 2 | Low | 0.715 | 0.178 | 7.785 |
| 1 | 2 | 0 | 1 | 4 | 6 | Low | 0.953 | 0.425 | 3.297 |
| 2 | 2 | 0 | 1 | 2 | 0 | High | Inf | - | Inf |
| 0 | 0 | 1 | 1 | 2 | 0 | High | Inf | - | Inf |
| 1 | 0 | 1 | 1 | 9 | 4 | High | **3.217** | **1.201** | **10.177** |
| 2 | 0 | 1 | 1 | 3 | 1 | High | 4.29 | 0.603 | 40.687 |
| 0 | 1 | 1 | 1 | 6 | 0 | High | Inf | - | Inf |
| 1 | 1 | 1 | 1 | 5 | 14 | Low | 0.511 | 0.299 | 1.375 |
| 2 | 1 | 1 | 1 | 0 | 4 | Low | 0 | - | - |
| 1 | 2 | 1 | 1 | 2 | 9 | Low | 0.318 | 0.164 | 1.442 |
| 2 | 2 | 1 | 1 | 1 | 3 | Low | 0.477 | 0.153 | 4.521 |
| 0 | 0 | 2 | 1 | 2 | 0 | High | Inf | - | Inf |
| 1 | 0 | 2 | 1 | 0 | 2 | Low | 0 | - | - |
| 2 | 0 | 2 | 1 | 0 | 1 | Low | 0 | - | - |
| 0 | 1 | 2 | 1 | 1 | 0 | High | Inf | - | Inf |
| 1 | 1 | 2 | 1 | 4 | 5 | High | 1.144 | 0.473 | 4.161 |
| 2 | 1 | 2 | 1 | 1 | 0 | High | Inf | - | Inf |
| 0 | 2 | 2 | 1 | 1 | 0 | High | Inf | - | Inf |
| 1 | 2 | 2 | 1 | 0 | 2 | Low | 0 | - | - |
| 1 | 0 | 0 | 2 | 2 | 2 | High | 1.43 | 0.356 | 9.994 |
| 0 | 1 | 0 | 2 | 1 | 0 | High | Inf | - | Inf |
| 1 | 1 | 0 | 2 | 4 | 2 | High | 2.86 | 0.712 | 15.334 |
| 2 | 1 | 0 | 2 | 1 | 2 | Low | 0.715 | 0.178 | 7.785 |
| 0 | 2 | 0 | 2 | 1 | 0 | High | Inf | - | Inf |
| 1 | 0 | 1 | 2 | 2 | 4 | Low | 0.715 | 0.267 | 3.834 |
| 2 | 0 | 1 | 2 | 1 | 0 | High | Inf | - | Inf |
| 1 | 1 | 1 | 2 | 4 | 0 | High | Inf | - | Inf |
| 2 | 1 | 1 | 2 | 3 | 0 | High | Inf | - | Inf |
| 1 | 2 | 1 | 2 | 0 | 4 | Low | 0 | - | - |
| 2 | 2 | 1 | 2 | 0 | 1 | Low | 0 | - | - |
| 1 | 0 | 2 | 2 | 0 | 1 | Low | 0 | - | - |
| 1 | 1 | 2 | 2 | 1 | 0 | High | Inf | - | Inf |
| X8: p53 (Arg^72^Arg): 0 - Arg/Arg; 1 - Arg/Pro; 2 - Pro-Pro X10: XRCC1 (Arg^399^His): 0 - Arg/Arg; 1 - Arg/His, 2 - His/His  X20: mEH (Tyr^113^His) : 0 - Tyr/Tyr, 1 -Tyr/His, 2 - His/His X1: 0 - No habit;  1 - Tobacco habit chewing, smoking or both;  2- Tobacco habit (chewing, smoking or both) with alcohol. | | | | | | | | | |
