# Supplementary material for: Multiple Pathway-Based Genetic Variations Associated with Tobacco Related Multiple Primary Neoplasms
Source: PLoS One. 2012 Jan 11;7(1):e30013. doi: 10.1371/journal.pone.0030013 (PMC3256192; doi:10.1371/journal.pone.0030013)
Supplement: Table S3 — Linkage Disequilibrium (LD) analysis. (DOC) [file pone.0030013.s003.doc]

# Supplementary Table S3: Linkage Disequilibrium (LD) analysis.

| No. | Combination | LD score | Chi-square |
| --- | --- | --- | --- |
| 1 | *XRCC1* Arg^194^Trp and *XRCC1* Arg^280^His | -0.008 | 0.15 |
| 2 | ***^#^XRCC1* Arg^194^Trp** and **^#^*XRCC1* Arg^399^Trp** | ****-0.037** | ****21.12** |
| 3 | *XRCC1* Arg^280^His and *XRCC1* Arg^399^Trp | 0.014 | 0.47 |
| 4 | *XRCC1* Arg^194^Trp and *XRCC3* Thr^241^Met | -0.007 | 0.93 |
| 5 | *XRCC3* Thr^241^Met and *XRCC1* Arg^280^His | -0.003 | 0.04 |
| 6 | *XRCC3* Thr^241^Met and *XRCC1* Arg^399^Trp | -0.010 | 0.29 |
| 7 | ****NAT2*  Ile^114^Thr** and ****NAT2* Arg^197^ Gln** | ****-0.079** | ****23.27** |
| 8 | ****NAT2*  Ile^114^Thr** and ***NAT2* Gly^286^Glu** | ****0.172** | ****9.69** |
| 9 | *NAT2* Arg^197^Gln and *NAT2* Gly^286^Glu | -0.017 | 3.60 |
| 10 | *****BRCA2* Asp^991^Asn** and *****BRCA*2 Asn^372^His** | ****-0.012** | ****20.17** |

## ^#^HWE; *Not in HWE; **LD

The linkage disequilibrium analysis: Combinations in bold showed significant association. The analysis was carried out using EH program.
